# Use of Microarray Datasets to generate Caco-2-dedicated Networks and to identify Reporter Genes of Specific Pathway Activity

**DOI:** 10.1038/s41598-017-06355-0

**Published:** 2017-07-28

**Authors:** Prashanna Balaji Venkatasubramanian, Gamze Toydemir, Nicole de Wit, Edoardo Saccenti, Vitor A. P. Martins dos Santos, Peter van Baarlen, Jerry M. Wells, Maria Suarez-Diez, Jurriaan J. Mes

**Affiliations:** 1 Wageningen University & Research, Food & Biobased Research, Bornse Weilanden 9, 6708 WG Wageningen, The Netherlands; 2 Alanya Alaaddin Keykubat University, Faculty of Engineering, Food Engineering Department, Kestel-Alanya, 07450 Antalya, Turkey; 3 Wageningen University & Research, Systems and Synthetic Biology, Stippeneng 4, 6708 WE Wageningen, The Netherlands; 4 LifeGlimmerGmbH, Markelstrasse 38, 12163 Berlin, Germany; 5 Wageningen University & Research, Host-Microbe Interactomics, De Elst 1, 6708 WD Wageningen, The Netherlands

## Abstract

Intestinal epithelial cells, like Caco-2, are commonly used to study the interaction between food, other luminal factors and the host, often supported by microarray analysis to study the changes in gene expression as a result of the exposure. However, no compiled dataset for Caco-2 has ever been initiated and Caco-2-dedicated gene expression networks are barely available. Here, 341 Caco-2-specific microarray samples were collected from public databases and from in-house experiments pertaining to Caco-2 cells exposed to pathogens, probiotics and several food compounds. Using these datasets, a gene functional association network specific for Caco-2 was generated containing 8937 nodes 129711 edges. Two *in silico* methods, a modified version of biclustering and the new Differential Expression Correlation Analysis, were developed to identify Caco-2-specific gene targets within a pathway of interest. These methods were subsequently applied to the AhR and Nrf2 signalling pathways and altered expression of the predicted target genes was validated by qPCR in Caco-2 cells exposed to coffee extracts, known to activate both AhR and Nrf2 pathways. The datasets and *in silico* method(s) to identify and predict responsive target genes can be used to more efficiently design experiments to study Caco-2/intestinal epithelial-relevant biological processes.

## Introduction

Biological networks are representational interactions between genes, proteins, and other biomolecules. Different kinds of biological networks (e.g protein-protein interaction or signalling networks) represent different features of a cell^1^. Such networks can be usefully exploited to gain key insights into biological systems^2, 3^. Exploration of tissue and cell type specific networks has demonstrated the effects of tissue specific regulation on the remodelling of biological networks^4^. Differential network analysis has also been used to compare topological characteristics of networks corresponding to normal or tumorous cells and to isolate characteristics of distinct cancer subtypes, which in turn has led to the prediction of cancer subtype-specific drug targets^5^. One important biological system is the epithelial cells lining the small and large intestine. The role of diet and the response of host towards diet and its compounds is challenging to be studied *in vivo* due to the complexity of biological systems and inter-individual variability. Thus, a reductionist approach using the human Caco-2 intestinal epithelial cell line is a widely accepted laboratory model to understand the response of intestinal enterocytes exposed to nutrition and microbes^6–8^. Although Caco-2 cells were derived from a colon carcinoma, when cultured as confluent monolayers for 2–3 weeks, they functionally resemble the enterocytes lining the small intestine^9^. Caco-2 cells have been used in numerous experiments to study effects of food products and compounds^6, 7, 10–13^, probiotics^8, 14^, pathogens^15–17^ and other studies^18–20^, using microarrays. Comparative proteomic analysis of Caco-2 cells and scrapings of the human intestinal epithelium support the usability of this *in* *vitro* model^21^, although Caco-2 cells appear to over-express as well as under-express certain proteins which needs to be considered in the interpretation of *in vitro* data and translation of results to the *in vivo* situation^21^.

A compendium of Caco-2 gene expression profiles under a broad number of conditions can be instrumental in building dedicated network models describing gene interactions in human intestinal enterocytes and in providing new insights on their functioning. Although, gene profiles tuned for selected tissues^22–24^ are present, to the best of our knowledge, no broad compendium of Caco-2 microarray experiments has been initiated, limited data on metabolic networks is available^25, 26^ and no gene/protein association networks are available for Caco-2/intestinal enterocytes. Another commonly faced problem is the identification of Genes Of Interest (GOI) in the pathways investigated for a specific cell type. Thus identification of candidate sets of GOI could help study the impact of treatments on specific pathways of interest in a given cell type.

Intestinal epithelial cells, apart from major functions like digestion and absorption of nutrients, minerals and water^27, 28^, play an important role in the exclusion or detoxification of xenobiotics and regulating oxidative stresses. The AhR and Nrf2 pathways are involved in the metabolism of xenobiotics and protection against oxidative stress^29, 30^. AhR is an important regulator of Phase I and Phase II enzymes and other enzymes which metabolize compounds such as dioxins, polycyclic aromatic hydrocarbons, plant polyphenols and tryptophan photoproducts^31^. Nrf2 has been designated the “master regulator” of the adaptive response to oxidative stress^29^ and regulates the expression of antioxidant proteins that protect against oxidative damage triggered by injury and inflammation.

In this study, we aim to i) exploit the knowledge accumulated in the publicly available datasets on Caco-2 cells exposed to different treatments in order to generate a dedicated network model accounting for gene associations specific to intestinal enterocytes and ii) to develop workflows to reliably select genes for studying intestinal enterocyte-specific pathways. The proposed strategies were experimentally validated by focussing on GOI in the Nrf2 and AhR pathways using Caco-2 cells exposed to coffee to induce the gene responses within these pathways. The obtained networks are provided as supplementary files (Caco2_Network) and R scripts for the identification of GOI are made available at http://semantics.systemsbiology.nl/index.php/download-page/ with a working example.

## Results

### Cell/Tissue-specific gene expression profiles aid the identification of reporter genes for specific pathway activity

In this study, we develop strategies to generate dedicated gene network models for Caco-2 and identify specific gene responses to nutrition related exposures. This was illustrated using Ahr and Nrf2 pathways. We have independently validated our results through a new experimental setup on which Caco-2 cells were exposed to coffee extracts, which have previously been shown to induce the Ahr and Nrf2 pathways^32^. Coffee extracts have a great chemical diversity and the components vary according to the cultivar, treatment, processing, storage and others^33–36^. We have tested induction of these pathways using four coffee types.

To identify reporter genes for the AhR and Nrf2 pathways, scientific literature was searched and we investigated whether these genes were also responsive to oxidative stress in our Caco-2 model after exposure to TCDD (2,3,7,8-Tetrachlorodibenzo*-p-*dioxin) or coffee. 16 genes that are frequently used as indicators for AhR and Nrf2 signalling, were selected from the literature (Table 1) for validation. Caco-2 cells were exposed to coffee extracts (Turkish coffee, Brasil Espirito, Java Preanger, Nescafe©) and TCDD and relative expression of the selected genes was measured by qPCR. Out of the 16 genes tested, 3 genes were not detectable (CT values ≥ 35) and 5 genes showed no differential expression (DE), a fold change threshold of 1.5 folds up or down in at least two of the coffee samples, indicating that 50% of the genes selected from literature are not useful for studying the activities of the AhR and Nrf2 pathways in enterocytes.

**Table 1:** Expression changes upon coffee/xenobiotics exposure of initial set of genes selected based on existing literature.

Gene Name	Pathway	Reference	Significant change in expression (Fold Change larger/smaller than ±1.5)
SQSTM1	Nrf2	Jain *et al*., 2010^68^	Yes
HMOX1	Nrf2	Bøhn *et al*., 2014^33^	Yes
Nrf2	Nrf2	Bøhn *et al*., 2014^33^	No
ABCC1	Nrf2	Adachi *et al*., 2007^69^	No
ABCC2	Nrf2	Adachi *et al*., 2007^69^	No
NQO1	Nrf2	Bøhn *et al*., 2014^33^	Yes
ABCG2	Nrf2	Isshiki *et al*., 2011^70^	No *
GSTP1	Nrf2	Steinkellner *et al*., 2005^71^	Yes
ARNT	AhR	Ishikawa *et al*., 2014; Yeager *et al*., 2009^32, 72^	No
AhR	AhR	Kalthoff *et al*., 2010^73^	Yes
CYP1A1	AhR	Ishikawa *et al*., 2014^32^	Yes
TiPARP	AhR	Diani-Moore *et al*., 2010^74^	Yes
UGT1A6	AhR	Yeager *et al*., 2009^72^	Yes
CYP1A2	AhR	Ishikawa *et al*., 2014^32^	Not detected
CYP1B1	AhR	Ishikawa *et al*., 2014^32^	Not detected
AHRR	AhR	Mimura *et al*., 2003; Abel *et al*., 2010^30, 31^	Not detected

‘*’Indicates genes found to be significantly differentially expressed (Fold change >±1.5) in Turkish Coffee only. Genes were considered to be responsive if they were expressed in at least two coffee samples.

### Compendium of Caco-2 experimental data supports cell-specific gene selection

A data compendium was generated using Affymetrix expression profiles of 341 arrays from 85 Caco-2 exposure experiments (Table 2). UPC filtering procedure was used to identify genes that are actively expressed in Caco-2 and 12849 genes were identified to be expressed. These genes were then used to generate a cell-specific network dedicated to Caco-2 intestinal epithelial cells.

**Table 2:** Summary of collected dataset.

Total Arrays	341
Total Experiments	88
From the lab of Jurriaan Mes	173
From Array Express	168
Type of Exposure	
Vegetables	9
Fruits	20
Fibres	22
Probiotics	7
Pathogens	11
Others	6
Food compounds	10

Supplementary Table S1 presents the comparison between network topological properties of the full interaction network retrieved from STRING (converted to Entrez Ids) and the Caco-2 specific network. The same cut-off (≥700) related to the reliability of the interactions (STRING combined score) was selected for both networks. The Caco-2 network is composed of 8937 nodes and 129711 edges and can be explored using common network visualization tools such as Cytoscape^37^. Notice the differences in the number of nodes and edges between the two networks.

Out of the 16 genes that we previously selected based on literature, ABCC1, ABCG2 and TIPARP are removed from the network of functional associations. This indicates that in the overall network they are connected only to nodes that show no (active) expression in our compendium. However, even after this reduction, still large number of genes remain (77 nodes for Nrf2 pathway and 42 nodes for AhR pathway) to probe for each pathway and therefore we wanted to optimize our approach to identify GOI.

### Biclustering analysis improves gene selection

The biclustering method works based on identification of genes that are co-expressed with seed genes (*i*.*e*. genes well known to be responsive in Caco-2 cells to a specific perturbation). In order to identify Caco-2 responsive genes within the Nrf2 pathway, we used a full list of genes that are involved in this pathway (derived from generic IPA consensus pathway). SQSTM1, HMOX1, NRF2, ABCC1, DNAJB1 and ENC1 were selected as seed genes. The seed genes were used to identify co-expressed genes within the compendium of microarrays. The initial average correlation threshold for array selection was set at 0.75 (default value). In this way, only arrays that showed a high degree of correlation with the seed genes were included for GOI identification.

The biclustering analysis reduced the 341 arrays (the initial number of arrays) to 229 arrays and the following genes were obtained as GOI: CDC34, DNAJC4, GTR, ATF4, GSTA2 and GSTM4. Together with the seed genes this resulted in a total of 12 potential responsive genes for the Nrf2 pathway (Table 3). These genes had an average correlation of 0.79 in the arrays included in this analysis.

**Table 3:** Expression changes upon coffee exposure of genes selected using the biclustering algorithm.

Gene Name	Pathway	Seed Genes	Found from	Significant change in expression (Fold Change more than ±1.5)
DNAJB1	Nrf2	Yes	WGCNA	—
SQSTM1	Nrf2	Yes	Literature	Yes
HMOX1	Nrf2	Yes	Literature	Yes
ENC1	Nrf2	Yes	WGCNA	No
Nrf2	Nrf2	Yes	Literature	No
ABCC1	Nrf2	Yes	Literature	No
CDC34	Nrf2	No	Biclustering	—
DNAJC4	Nrf2	No	Biclustering	—
GTR	Nrf2	No	Biclustering	—
ATF4	Nrf2	No	Biclustering	Yes
GSTA2	Both	No	Biclustering	Yes
GSTM4	Both	No	Biclustering	Yes
MAPK8	AhR	No	Biclustering	—
MED1	AhR	No	Biclustering	—
NCOR2	AhR	No	Biclustering	—
NFIA	AhR	No	Biclustering	No
ARNT	AhR	Yes	WGCNA	No
AhR	AhR	Yes	Literature	Yes
CYP1A1	AhR	Yes	Literature	Yes
PRKCA	AhR	Yes	WGCNA	No
TiPARP	AhR	Yes	Literature	Yes

‘—’ Indicates genes that were not the target of experimental validation. Genes were considered to be responsive if they were differentially expressed in at least two coffee samples.

Similarly, CYP1A1, TIPARP, AHR, ARNT and PRKCA were chosen as seed genes for AhR pathway. Owing to the small number of seed genes, mean correlation threshold for array selection was set at a more stringent value of 0.8. The biclustering analysis reduced the initial 341 arrays to 274 arrays and predicted GSTA2, GSTM4, MAPK8, MED1, NCOR2 and NFIA as GOI for the AhR pathway. This procedure reduced the number of potential responsive genes to 11 for AhR pathway (Table 3), including seed genes.

**Figure 1:**
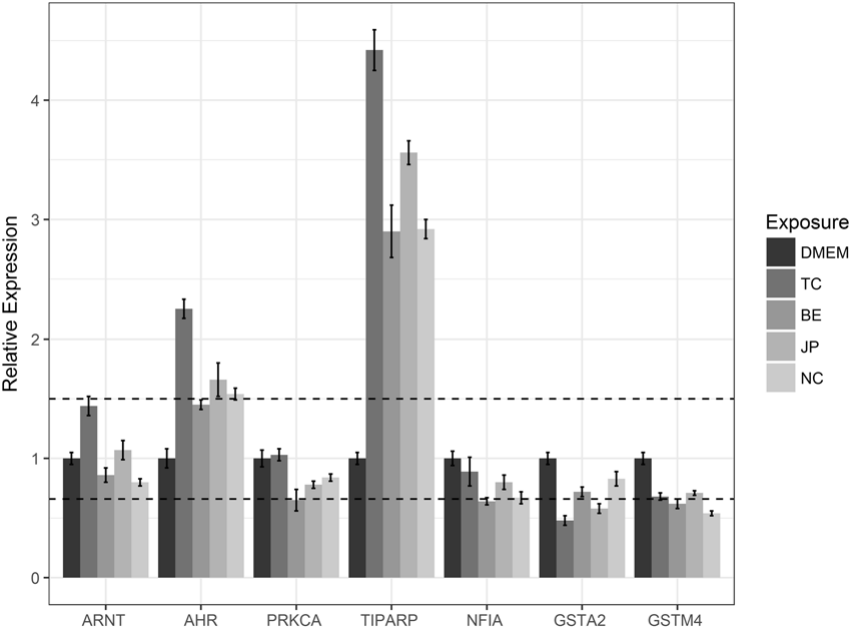
qPCR results for AhR Pathway genes predicted using biclustering algorithm. The plot shows the relative gene expression level (control vs treatment) of several genes associated with AhR pathway. Results have been normalized to control (DMEM) values. Values and error bars represent average and standard deviation of three replicates. Dashed lines represent the fold change cut-off limits (1.5 for up regulation and 0.6 for down regulation). CYP1A1 is not shown here as it exceeds the plot limits. TC indicates Turkish coffee, BE indicates Brasil Espirito, JP indicates Java Preanger and NC indicates Nescafe©.

**Figure 2:**
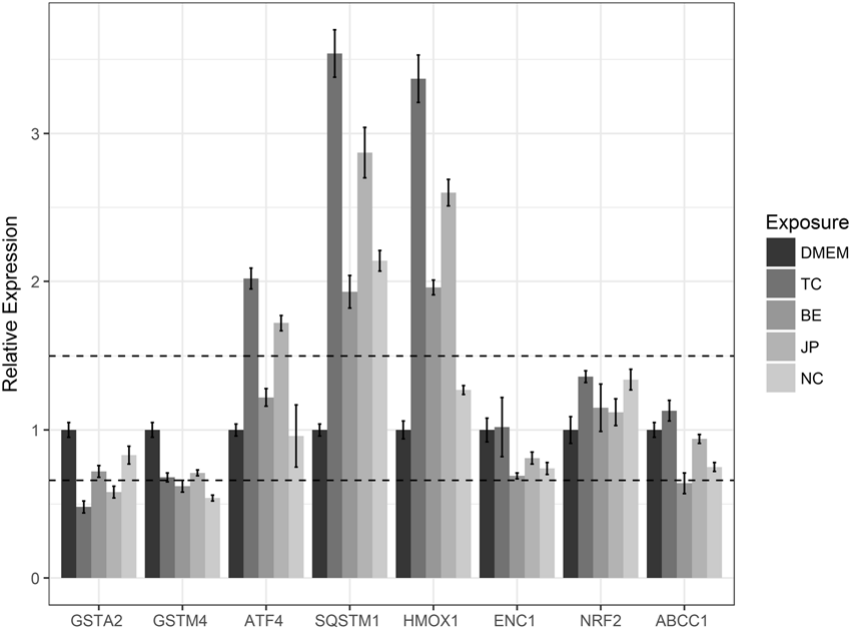
qPCR results for Nrf2 Pathway genes predicted using biclustering algorithm. The plot shows the relative gene expression level (control vs treatment) of several genes associated with Nrf2 pathway. The line represents the fold change cut-off limits (1.5 for up regulation and 0.6 for down regulation). TC indicates Turkish coffee, BE indicates Brasil Espirito, JP indicates Java Preanger and NC indicates Nescafe©.

We selected 14 genes for experimental verification using Caco-2 cells exposed to coffee extracts (Figs 1 and 2). Of these, 6 genes were specific to AhR pathway, 6 specific to Nrf2 pathway and 2 common to both pathways. Four of these genes have been predicted by the algorithm (“Biclustering” see Table 3). All 4 genes were found to be expressed in Caco-2 cells of which 3 showed substantial changes in expression (Fold Change > 1.5) between control and treatment (Figs 1 and 2).

Based on these results, we concluded that this strategy constitutes a useful addition to the literature data for gene selection. Selected genes extracted from the literature can be combined with the ones selected using the proposed approach. In those cases where literature provides an ample list of genes for experimental validation, our approach serves to further refine the selection of genes which are differentially expressed by Caco-2 cells in a chosen pathway.

### Differential Expression Correlation Analysis (DECA) further enhances gene selection

An assessment of DECA algorithm was performed using 10 pathways from the KEGG database^38^ that are of interest to intestinal epithelia. For each pathway 10 runs were performed using three randomly selected genes from the pathways as seed genes. Genes known to be in the target pathways were found to be significantly better ranked than genes not in the pathway, as indicated by the enrichment p-values. On average ~9% of genes related to each pathway could be predicted as target genes on analysing the top 10% ranked genes using DECA algorithm. The performance of the algorithm varied according to the pathway from 6% to 15%. This result indicates that without any further literature considerations DECA is able to retrieve genes associated to the pathway. In this assessment seed genes were chosen at random, however careful selection of seed genes is required to obtain more reliable prediction of target genes. As in the previous case, this approach would work best when combined with pre-existing knowledge. The results of the *in silico* assessment are provided in Supplementary Table S2.

The DECA method was applied to find a global set of genes (amongst all genes expressed in Caco-2) associated with Nrf2 and AhR pathways which are responsive to altered pathway activity. SQSTM1, NQO1 and HMOX1, involved in the Nrf2 pathway were used as seed genes for the DECA algorithm. 2834 genes were found to have correlation values or significance fractions above the 0.6 threshold against each seed gene. The genes were ranked as mentioned in Materials and Methods section and top ranked genes were considered for further analysis. From this list, GCLM^39^, TXNRD1^40^, SOX9 and KCTD5^41^ were selected for further experimental validation via qPCR as there is some evidence of involvement in this pathway. In addition, BAG3^42^ gene which did not belong to the top ranking genes was randomly chosen as a negative control (Table 4).

**Table 4:** Expression changes upon coffee exposure of genes identified using the DECA algorithm in AhR and Nrf2 pathways.

Gene Name	Pathway	Type	Significant change in expression (Fold Change more than ± 1.5)
CYP1A1	AhR	Seed Genes	Yes
TIPARP	AhR	Seed Genes	N/A
ATP9A	AhR	Predicted	No
UGCG	AhR	Predicted	Yes
CHMP1B	AhR	Predicted	Yes
EREG	AhR	Predicted	Yes
RND3	AhR	Predicted	Yes
SQSTM1	Nrf2	Seed Genes	Yes
HMOX1	Nrf2	Seed Genes	N/A
NQO1	Nrf2	Seed Genes	N/A
BAG3	Nrf2	Predicted	No *
SOX9	Nrf2	Predicted	Yes *^
TXNRD	Nrf2	Predicted	Yes
GCLM	Nrf2	Predicted	Yes
KCTD5	Nrf2	Predicted	Yes *^

‘*’Indicates genes found to be significantly differentially expressed (Fold change > ± 1.5) in Turkish Coffee only. ‘^’Indicates genes found to be significantly differentially expressed (Fold change > ± 1.5) in Nescafe only. N/A indicates genes that were not the target of experimental validation. Genes were considered to be responsive if they were expressed in at least two coffee samples.

A similar approach was used to predict the GOI in the AhR pathway. Only two genes, CYP1A1 and TIPARP were chosen as the seed genes for the DECA algorithm which resulted in a list of 398 ranked genes. From this list, UGCG^43^, EREG^44^, RND3, CHMP1B were chosen for experimental verification as evidence from scientific literature associated few of them with the AhR pathway. ATP9A was randomly selected as a negative control (Table 4).

**Figure 3:**
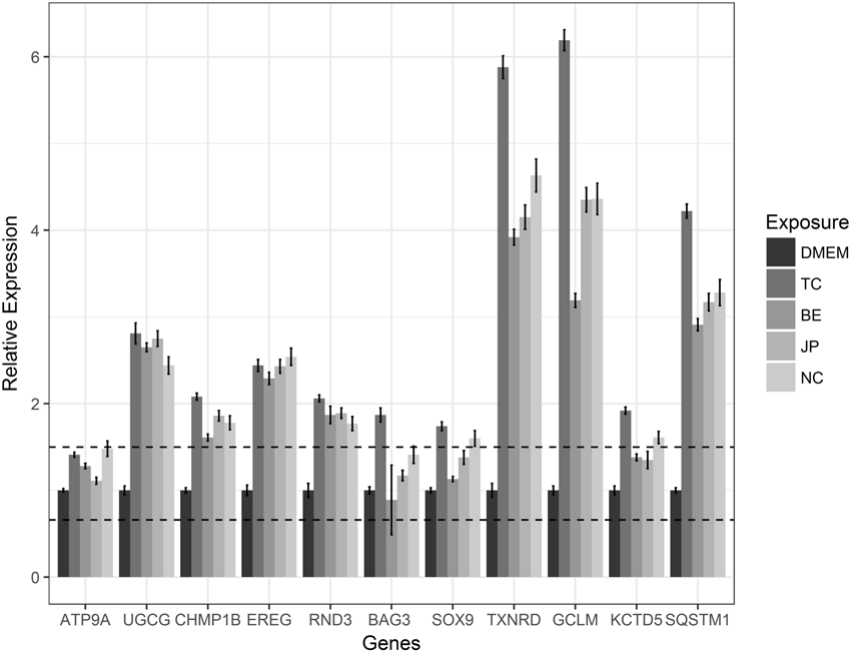
qPCR results of both AhR and Nrf2 pathways provided together for genes predicted using DECA algorithm. The line represents the fold change cut-off limits (1.5 for up regulation and 0.6 for down regulation). CYP1A1 is not shown here as it exceeds the plot limits. TC indicates Turkish coffee, BE indicates Brasil Espirito, JP indicates Java Preanger and NC indicates Nescafe©.

The above mentioned 10 genes along with a seed gene for each pathway were experimentally verified using qPCR analysis in Caco-2 cells exposed to coffee samples (Fig. 3). The results indicate that 75% of the selected GOI showed a substantial relative difference in expression (absolute fold change > 1.5) in all tested samples, 2 genes (SOX9 and KCTD5) were differentially expressed upon exposure to two of the coffee extracts (Turkish and Nescafe, absolute fold change > 1.5) while the control genes showed no significant change in expression in most coffee extracts, as expected.

These results indicate that the DECA is a substantially improved strategy to identify GOI compared to other methods discussed in this paper and moreover does not require prior knowledge of the genes within the pathway except for the seed genes.

## Discussion

Initially we focussed on developing an intestinal enterocyte-specific association network using expression data from Caco-2 cells exposed to different nutrients and stimuli. The network was constructed by selecting 12849 genes (actively) expressed in Caco-2 based on UPC filtering. This is consistent with previous observations of 11559^26^ and 14113 genes^24^ based on RNAseq data (Caco-2 cells grown under controls). Differences could be attributed to different selection procedures or experimental approaches. Additionally, the gene list and network provided in this paper are based on a compendium of transcriptomics data from exposure of Caco-2 cells to different nutrients and stimuli.

When applying our Caco-2-specific selection to STRING network the number of edges and nodes was reduced considerably (~50%). The number of connected components is reduced by over 60% and the local network structure is preserved with similar values of clustering coefficient, which suggests a more compact network, as expected for gene that are functionally closely related. The degree assortativity decreases indicating less redundancy on gene associations when the network is restricted to Caco-2. Incidentally STRING could support dedicated data analysis by enabling seamless tissue specific gene selection.

Biclustering simultaneously clusters both genes and samples to arrive at the identification of genes with similar expression profiles in a subset of the samples. Existing biclustering algorithms do not allow targeting a particular pathway^45, 46^, instead they generally try to find biclusters that cover either a broad range of genes or conditions. Similarly WGCNA based clustering does not focus on a particular pathway but looks for modules of co-expressed genes that may belong to more than one pathway. Here we present a biclustering approach, that represents a modification of that in van dam *et al*., that allows the user to select or pre-select the seed genes and thus a pathway^47^. Nevertheless, biclustering performed poorly as the identified GOI did not show significant DE, indicating little responsiveness of Caco-2 cells to coffee exposures.

Therefore, DECA algorithm was used, resulting in a list of responsive gene candidates and a set of criteria to further rank them. From the ranked list, genes were selected for experimental verification in Caco-2 cells exposed to coffee and we found association with AhR and Nrf2 pathways. The verified genes were not in these pathways as defined in IPA. It might be that some of these genes have an indirect association to these pathways. The DECA ranking can be combined with existing knowledge, for instance, adding weight to genes on the basis of literature evidence. Of the 5 genes predicted for Nrf2 pathway, GCLM and TXNRD1 are previously known downstream gene targets of NRF2^39, 40^. KCTD5 is likely to have an indirect interaction mediated by CUL3^41^ and BAG3 (negative control gene) has been associated with Nrf2 pathway^42^ while we find that only Turkish coffee induces this gene. Similarly for the genes predicted for AhR pathway, UGCG is indirectly linked to AhR pathway via ARNT^43^ and EREG is reported as a target gene for AHR^44^.

Seed genes play a critical role in predicting responsive genes in a certain pathway and should be carefully considered and accurately selected. As an example, Nrf2 gene was initially included among the seed genes for the biclustering algorithm. However, experimental verification showed transcript levels of this gene not to be responsive to coffee exposure. It was later not used as seed gene for DECA algorithm and was replaced with NQO1. One optimal way to select seed genes is to select two or three highly differentially expressed genes (Fold Change > 3) associated to the pathway of interest from literature (eg. CYP1A1 and TIPARP for AhR pathway), verify their altered expression in response to activation or repression of the pathway and use these as seed genes.

The biclustering algorithm requires a further selection of genes to be considered, the gene pool set. This selection was performed by aggregating non cell type specific pathway level information. On the other hand, DECA has no such constraint and the whole set of expressed genes are considered. Therefore DECA is our method of choice to identify GOI in pathways for which little information is available. One could also argue that, when combining such a large set of array data collected over different batches, batch correction techniques should be applied. However, here each experiment has its own control in the same batch. As a result batch effects and experimental effects might be confounded and usually applied correction methods such as ComBat and SVA are not effective^48, 49^. Instead, we have used a higher level integration approach, in which data from each study is compared with the corresponding control. This way we bypass the need for additional batch corrections as we study only correlations between changes in gene expression.

In addition to predicting GOI, the compendium presented in this paper can be used for other purposes. For instance, a systematic categorization of the treatments based on expression profile, similar to the approach taken in Connectivity map^50^ and thus could select food components that have effects on certain genes and pathways. Such datasets can also be used to predict key regulators and/or gene hubs^2^. Additionally, the database can be expanded further by adding data from future experiments, even from technologies like RNAseq. The provided Caco-2 specific network also serves as a platform to understand future experiments. Gene expression data from a new experiment could be integrated with this network by using algorithms for network mining and active module identification^3^. The Caco-2 cell type specific network can also be used to develop networks associated to different conditions such as Caco-2 exposure to pathogens or pathogenic toxins, then these networks can be used to identify potential drug targets by applying statistical methods and identifying hub genes using similar strategies as the one successfully used in cancer research^51^. This paper can therefore be seen as a first important step to improve current analysis tools for Caco-2 and thereby elicit a better understanding of the interaction between our intestinal epithelium and luminal (nutritional) compounds.

## Conclusion

Caco-2 cell lines are increasingly used as model systems to study the interaction of food and other luminal factors with the intestinal system of the host, which is difficult to study *in vivo*. As the availability of experimental datasets will grow further we believe that this work is the first step in generation of a Caco-2 specific database and tissue specific research tools and strategies to extract more knowledge from these data. One of the research tools for which we make an important step is the dedicated protein-protein association network using gene expression data for Caco-2. The network provided in this paper could be the basis to be implemented in other software tools like IPA and STRING and can be further updated when more data become available in the future. The modified biclustering and DECA methods should additionally provide the necessary tools to extract genes of a desired pathways and can be applied, by the codes provided, to a similar dataset of any cell type of interest.

In the future, a comprehensive Caco-2 transcriptome database should include microarray data from other platforms such as Agilent, Illumina, etc but more importantly should include RNAseq data which will provide additional information on splice isoforms. We believe that such a cohesive database would provide finer results regarding the genes of interest in Caco-2 and can support the analysis and understanding of future Caco-2 cell based analysis. The dataset can additionally be used for building classifiers using genetic profiling and in finding therapeutic food solutions.

## Materials and Methods

### Data Processing

Caco-2 microarray gene expression data were obtained from public repository, Array Express (www.ebi.ac.uk/arrayexpress) and from in-house experiments performed using Affymetrix^©^ 1.1 ST array platform. In-house data was obtained by exposure of Caco-2 cells grown on transwells with different preparations of food-related compounds in experiments conducted over several years. Publicly available data was restricted to experiments on Affymetrix platform. Data and associated metadata were manually curated using the following inclusion criteria: i) experiments that did not induce genetic mutations, ii) experiments performed on Caco-2 cell monolayers that were grown for at least seven days and iii) arrays probing for at least 17000 genes (annotated in Chip Definition Files), thereby leaving out old arrays. Based on these criteria 341 arrays were selected corresponding to 22 experimental batches encompassing 85 different treatments (Table 2). GSE accession numbers of publicly available datasets and other relevant descriptions are given in Supplementary Table 3.

The consolidated data of 341 arrays were normalized using the SCAN algorithm before network construction and biclustering analysis, as this is a method that performs well for cross comparison^52^. RMA normalization was used for differential expression (DE) analysis, as it is considered as standard for this calculation^53^. All the normalization procedures were performed using R Bioconductor packages *SCAN*.*UPC* ^54^ and *affy* ^55^. Microarray probes were matched to gene identifiers using the CDF array annotation (version 18) provided by the University of Michigan microarray© lab^56^. After both normalization procedures, a combined set of 21996 genes was obtained. All statistical programming were performed using statistical language R (version 3.2.3).

### Identification of genes expressed in Caco-2 cells

Universal exPression Code (UPC) was used to obtain a standardized score describing the active/inactive state of each gene in each array of our data compendium^54^. Genes with a UPC value greater than 0.5 in at least one array were considered to be expressed in Caco-2 cells and therefore used in the analysis. This step was applied to the matrix of 21996 genes and 341 arrays reducing it to a matrix of 12849 genes and 341 arrays. In this matrix there were some genes with some values missing, likely due to platform differences. Therefore, genes with missing values in more than half the total number of arrays (*ie*. 170 arrays) were discarded. Remaining missing values were imputed using KNN algorithm from the ‘impute’ R package in refs 57 and 58 with default parameters. The final data matrix contained values for 10831 genes over 341 arrays.

### Caco-2 cell specific network generation

The database STRING (version 10)^59^ was used for the retrieval of high confidence human specific protein association and a combined score cut-off value of 700 was used as recommended by STRING. Nodes representing genes identified as not being expressed by Caco-2 cells were removed from the network. The network (in edgelist format) is available as supplementary file (Caco2_Network). Edgelist contains pairs of interacting genes (first two columns) and in this file genes are denoted by their Entrez Ids. The third column refers to the weight of each edge, which is however empty in the given file, as the edges have no weights. The networkx (python package) was used for network topological analysis^60^.

### Biclustering Algorithm

The Biclustering algorithm of cMonkey^45^ adapted by van Dam *et al*.^47^ was used to find biclusters (*i*.*e*. groups of co-expressed genes in a subset of conditions^61, 62^). In our implementation a pre-defined set of genes, called seed genes, together with additional genes from a second list called gene pool were used to find biclusters. Seed genes were selected using the following two approaches: i) from literature on Caco-2 expression in response to different types of coffee (SQSTM1, HMOX1, NRF2 and ABCC1 for the Nrf2 pathway and CYP1A1, TIPARP and AHR for AhR pathway). ii) from Weighted Gene Correlation Network Analysis^63^ (WGCNA). The WGCNA method partitions genes expressed in Caco-2 cell lines into groups enriched for topological overlap based on their expression profiles. These groups were then assessed for enrichment in genes belonging to the selected pathways using Ingenuity Pathways Analysis (IPA) (http://www.ingenuity.com, release March 2014). Genes assigned to the selected pathways in the enriched modules (FDR < 0.05) were further included in the seed gene list (DNAJB1 and ENC1 for Nrf2 pathway and ARNT and PRKCA for AhR pathway). To build the gene pool, genes expected to be in the pathway of interest were retrieved from pathway database IPA (Ahr and Nrf2 consensus pathway).The gene pool list contained 87 genes for Nrf2 pathway and 48 genes for AhR pathway.

**Figure 4:**
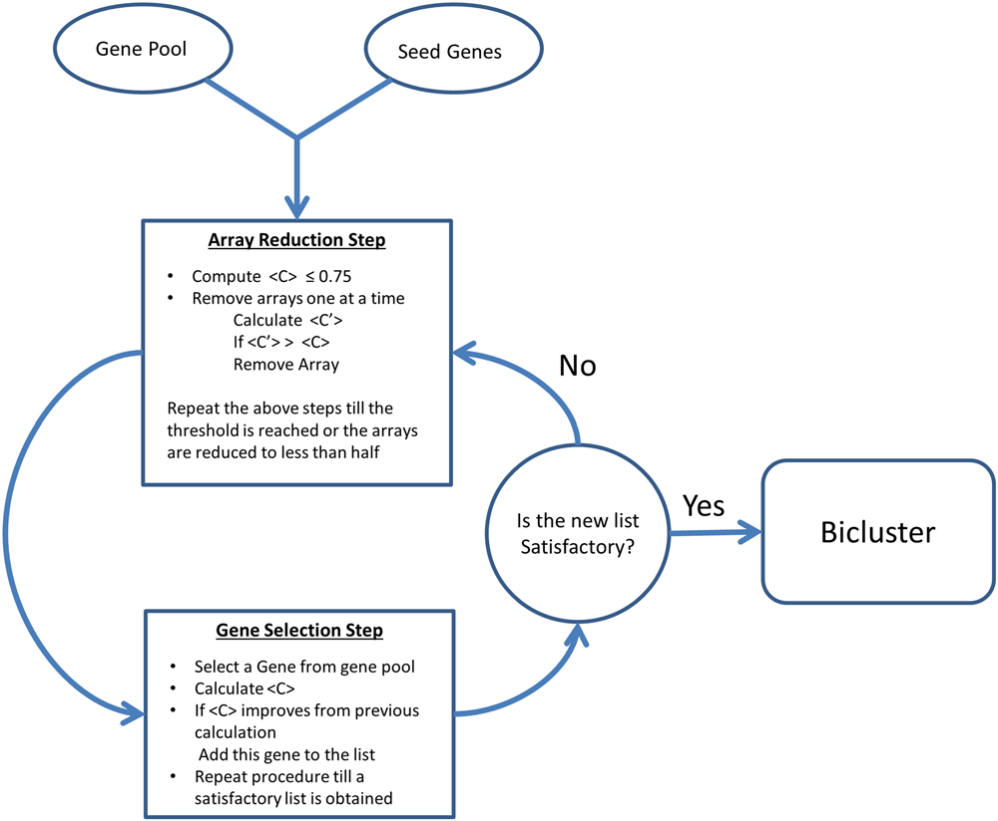
Flow diagram describing Biclustering algorithm. Seed genes are a predefined group of genes. The gene pool is the set of genes to be tested for inclusion in the bicluster. <C> indicates mean pairwise correlation, <C′> indicates new mean pairwise calculation.

Biclustering was performed using R implementing the iterative procedure depicted in Fig. 4. In the first step, the data compendium is explored to select arrays for which the seed genes show a high degree of mean pairwise correlation between each other. This selection is performed by iteratively removing one array from the list and comparing the average pairwise correlation between seed genes computed considering the full array list and the array list without the selected one. If removal of the considered array leads to an increase of this correlation, the array is permanently removed from the array list. This process is iterated until either the average correlation between seed genes is greater than or equal to a threshold value, C_T_ = 0.75 or half of the initial arrays have been removed.

Once the reduced array set has been established, an additional iterative procedure to search for candidate genes is performed. In the initialisation step, a new list of genes is built containing the seed genes. Then a new gene is selected from the gene pool and the mean correlation between this new gene along with the genes in the current list is calculated. If such correlation value is greater than previous correlation value, the new gene is added. This procedure is iterated till no new genes remain. The full procedure of array reduction and gene addition is continued until a bicluster with the desired properties is obtained.

### Differential Expression Correlation Analysis (DECA)

We implemented a new algorithm, Differential Expression Correlation Analysis (DECA) to find GOI using DE values from microarray datasets. The DECA algorithm works by calculating correlation values between seed genes and other DE genes identified using the UPC algorithm. DE values were calculated for 85 experimental setups (3 of which could not be used as they lacked sufficient replicates or controls) giving a total of 21996 genes. For each of these genes the treatments were compared to their respective controls using Bioconductor package *limma* ^64^. Following this, UPC filtering was applied and the DE matrix (a matrix containing the DE values with genes along the rows and experimental comparisons along the columns) was reduced to 12849 genes. Genes that were missing expression values for more than 56 conditions (roughly two third conditions) were excluded and then remaining missing data were imputed using KNN impute as mentioned above. This resulted in a matrix of DE values for 12462 genes and 85 conditions. All corresponding missing p-values were substituted with 1.

The next step in DECA is the selection of seed genes from literature. Seed genes were chosen in such a way that they showed strong and significant (absolute fold change ≥2 and p-value < 0.01) DE in stimulations associated to the chosen pathway (SQSTM1, NQO1 and HMOX1 for the Nrf2 pathway and CYP1A1 and TIPARP for AhR pathway).

**Figure 5:**
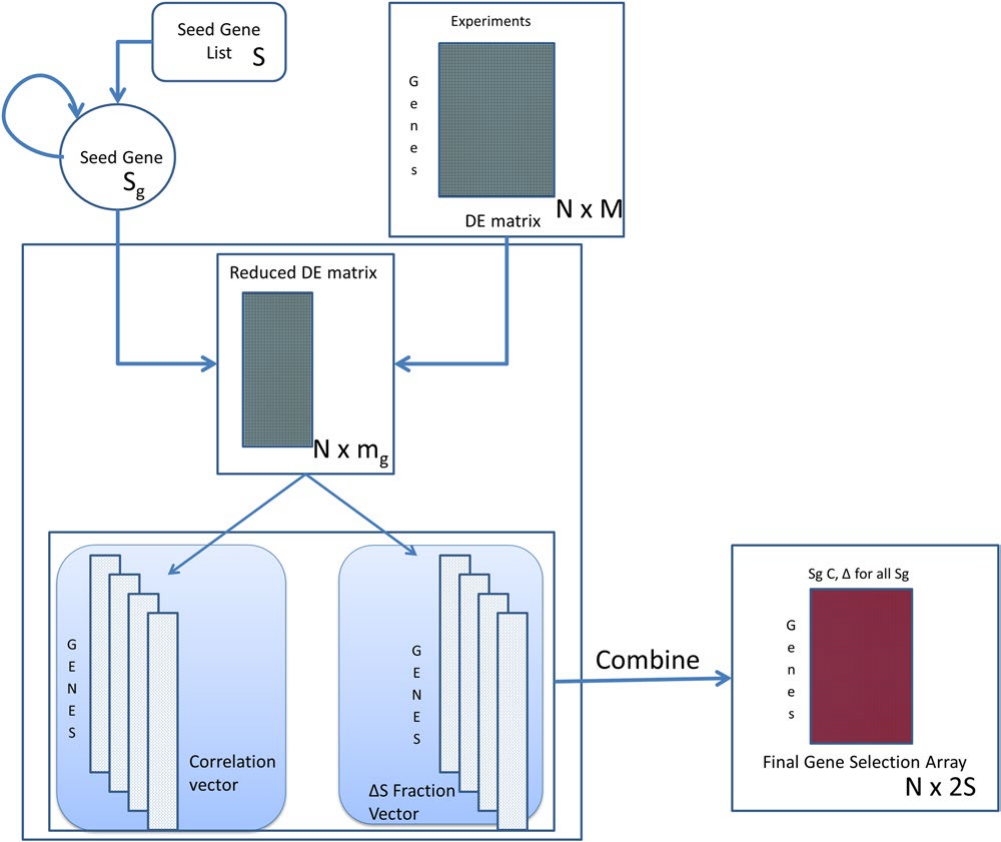
Flow diagram describing DECA (Differential Expression Correlation Analysis). Seed gene list refers to the starting gene selection. DE matrix is the input data matrix. The algorithm outputs a ranked list of genes which are highly correlated with the input genes.

The workflow of the procedure is described in Fig. 5 and implemented in R. Seed genes were then randomly considered one at a time. The DE matrix is reduced by the algorithm to contain only the comparisons in which the seed gene under consideration is found to have significant DE. Correlation values are calculated between the seed gene and each gene in the gene pool using the reduced DE matrix. The fraction of reduced comparisons in which each gene has significant DE (p-value < 0.01) is recorded and is termed significance fraction. Finally, correlations and fractions for each seed gene, are combined in a matrix format and a selection criterion for absolute correlation values and significance fraction was set at 0.6. A list of genes that have either absolute correlation value or significance fraction above the threshold for any of the seed gene is selected. Subsequently, this new list of genes is ranked depending on their individual absolute correlation values and significance fraction for each seed gene, thereby providing *2n* ranks (where *n* is the number of seed genes). A final rank was calculated by estimating the geometric mean of the 2*n* ranks for each gene.

All R scripts used in this paper are available at http://semantics.systemsbiology.nl/index.php/download-page/.

### DECA comprehensive *in silico* assessment

10 pathways were chosen at random for assessment of DECA algorithm. These pathways are ABC transporters pathway, Adherens junction pathway, Fat Absorption pathway, Gap junction pathway, Glycerolipid metabolism pathway Glycerophospholipid metabolism pathway, Nfk-β signalling pathway, p53 signalling pathway, PPAR signalling pathway and TLR signalling pathway. Some of these pathways are known to be associated with intestinal epithelia^65–67^. The genes associated to each of the 10 pathways were selected form KEGG pathway database^38^. For each of these pathways, 3 seed genes were chosen at random. The chosen seed genes were ensured for significant differential expression in at least 15 experiments. The seed genes were then used in DECA and the resulting gene list was ranked as mentioned above. The number of genes present in the top 10% of the ranked list belonging to the pathway were calculated. In addition to this, a Welch two sample t-test was performed to assess if the average ranks of the pathway related genes had a better rank compared against the average ranks of the rest of the genes in the ranked list. The protocol was iterated 10 times for each pathway. The results are provided in Supplementary Table S2.

### Culturing & experimental exposure of Caco-2 cells

The Caco-2 cells were cultured for 7 days until they reach confluence in DMEM (Dulbecco’s Modified Eagle Medium) (Control media) prior to exposure to coffee extracts (Turkish coffee [TC], Brasil Espirito [BE], Java Preanger [JP], Nescafe© [NC]) or TCDD. The RNA was harvested and primers were developed for qPCR. The detailed description of the protocol is provided in Supplementary Text F1.

### Data availability statement

The sources of datasets analysed during the current study are listed in Supplementary Table S3. Datasets that are not publicly available are available from the corresponding author on reasonable request.

## Electronic supplementary material

Supplementary Table S1

Supplementary Table S2

Supplementary Table S3

Supplementary Text F1

Caco2_Network
