# Supplementary material for: Use of Microarray Datasets to generate Caco-2-dedicated Networks and to identify Reporter Genes of Specific Pathway Activity
Source: Sci Rep. 2017 Jul 28;7:6778. doi: 10.1038/s41598-017-06355-0 (PMC5533711; doi:10.1038/s41598-017-06355-0)
Supplement: Supplementary file 4 — Supplementary Text F1 [file 41598_2017_6355_MOESM4_ESM.pdf]

Use of Microarray Datasets to generate Caco-2-dedicated Networks and to identify Reporter Genes of Specific Pathway Activity

Author list

Prashanna Balaji Venkatasubramanian, Gamze Toydemir, Nicole de Wit, Edoardo Saccenti, Vitor A. P. Martins Dos Santos, Peter van Baarlen, Jerry M. Wells, Maria Suarez-Diez and *Jurriaan J. Mes.

The following is a part of the supplementary information of the publication titled above.

Culturing & experimental exposure of Caco-2 cells

The Caco-2 cell line was obtained from the American-Type Culture Collection (ATCC HTB-37TM; USA). The cells were routinely grown in 75 cm^2 tissue culture flasks (with canted neck and 0.2 µm vented cap, Corning, 430641) using Dulbecco's Modified Eagle's Medium (DMEM) (Invitrogen, 42430-082; with 4.5 g/L glucose, no pyruvate, 4 mM L-glutamine, and 25 mM HEPES) supplemented with 9.1% Fetal Bovine Serum (FBS, Hyclone Perbio) (Fischer Scientific CH 30160.03; heat inactivated at 56ºC for 45 min). Cells were maintained in a humidified atmosphere of 5% CO_2 in air at 37ºC and sub-cultured at 80-90% confluence. For exposure experiments, Caco-2 cells were grown on transwells (Greiner bio-one, 662640, translucent, 0.4 µm pores, $1x10^8$ pores/cm, 0.312 cm^2 surface area for cell growth) in 24-well plates (Greiner bio-one Cellstar plates, 662102, Alphen a/d Rijn, The Netherlands). Cells, having a passage number between 30 and 45, were seeded at a concentration of $0.225x10^6$ cells/mL and grown in DMEM supplemented with 10% FBS, at 37ºC and 5% CO_2 in air. Cells were allowed to grow for 7 days and the culture medium was replaced every two days. To ensure that the monolayers exhibit the properties of a tight biological barrier, transepithelial electrical resistance (TEER) was monitored using a MilliCell-ERS voltohmmeter (Millipore Co., United States). Monolayers with TEER values exceeding 300 $\Omega.cm^2$ were used exclusively for the experiments.

Turkish coffee (obtained from a local market in Turkey), 2 types of filtered coffees (Java Preanger and Brasil Espirito) (obtained from a local market in The Netherlands), and instant coffee (Nescafe Gold Blend obtained from a local market in The Netherlands) samples were brewed without any sugar and/or milk addition. For Turkish coffee brew, 10 g of ground coffee sample was cooked with 130 mL of MQ water until the boiling point (53). For the filtered coffee brews, 8 g of powder was extracted with 140 mL of boiled MQ water (54) and for the instant coffee brew, 2 g of instant coffee was solubilized in 150 mL of boiled MQ water (55). All coffee brews were first filtered (Whatman Filter Paper, 589/1, ashless, Whatman, U.K.), 2 times, and then freeze-dried, and stored at -80ºC until analysis.

For the Caco-2 cell exposure experiments, the freeze-dried coffee extracts were re-dissolved in cell culture medium (DMEM with 9.1% FBS) to give the same dry-weight concentrations as in the original coffee brews. For the sample treatments, the culture medium was first removed from the well, and then coffee samples were added, in duplicates, to the apical side of the cells, with a volume of 150 µL; while the basolateral side was refreshed with culture medium only, with a volume of 700 µL. Cells

were incubated with samples for 24 h at culture conditions. During sample incubations, the cell monolayer integrity was checked with TEER measurements. After completion of the exposure experiments, the cells were harvested for RNA extraction.

After exposure, total RNA was isolated from the Caco-2 cells by using 200 µL of TriZol (Invitrogen, 15596-026, Paisley, UK). The TriZol extracts were subsequently treated with DNase (Qiagen, RNase free DNase set, #79254, Hilden, Germany), and purified with RNeasy mini columns (Qiagen, Hilden, Germany), using the protocol supplied by the manufacturer. The concentration and purity of the RNA samples were determined spectrophotometrically using a NanoDrop (ND-1000 Spectrophotometer, Thermo Fisher Scientific, Wilmington, USA).

One microgram of total RNA was reverse transcribed into cDNA using iScript^{TM} cDNA Synthesis Kit (Bio-Rad, Hercules, CA, USA) in a final volume of 20 µL. Primers used for the amplification of reference genes (β-actin, GAPDH, RPLP0) and the target genes (AhR, ARNT, CYP1A1, TiPARP, ABCC1, ABCC2, ABCG2, Nrf2, NQO1, GSTP1, GSTM4, GSTA2, UGT1A6, HMOX-1, SQSTM-1, ATF4, NFIA, PRKCA, ENC1 (NRPB), UGCG, EREG, RND3, CHMP1B, ATP9A, GCLM, TXNRD1, SOX9 KCTD5, BAG3) are given in Table S1 provided below.

qPCR amplification was performed with 5 µL diluted (40 times diluted) cDNA sample, 2.5 µL of each primer (3.2 µM for CYP1A1; 0.8 µM for the other primers) and 10 µL of SYBR Green Supermix (Bio-Rad, Cat# 172-5006CUST, Hercules, USA) in a final volume of 20 µL. Every sample was run in technical duplicates. Gene expression analysis was conducted on a BioRad CFX96 Real-Time System with C1000 Thermal Cycler. Gene expression levels were calculated using Biogazelle qbase^{plus} (Zwijnaarde, Belgium) program and the expression values of the selected target genes were normalized using the reference genes β-actin, RPLP0, and GAPDH (Table, provided below)

Table: Details of the gene names that were used in qPCR experiments and their corresponding Primers

Gene Name	Gene Symbol	Forward Primer (5¢-3¢)	Reverse Primer (5¢-3¢)
Beta Actin	ACTB	CTGGAACGGTGAAGGTGACA	AAGGGACTTCCTGTAACAATGCA
Glyceraldehyde-3-phosphate dehydrogenase	GAPDH	TGCACCACCAACTGCTTAGC	GGCATGGACTGTGGTCATGAG
Ribosomal protein, large, P0	RPLP0	GCAATGTTGCCAGTGTCTG	GCCTTGACCTTTTCAGCAA
Aryl hydrocarbon receptor	AhR	ACATCACCTACGCCAGTCG	CGCTTGGAAGGATTTGACTTGA
Aryl hydrocarbon receptor nuclear translocator	ARNT	GGAACAAGATGACAGCCTAC	CAGAAAGCCATCTGCTGCC
Cytchrome P450, family 1, subfamily A, polypeptide 1	CYP1A1	TCTTTGGAGCTGGGTTTG	ACTGTGTCTAGCTCCTCTTG
TCDD-inducible poly (ADP-ribose) polymerase	TiPARP	AGAACGAGTGGTTCCAATCCA	TGGGTGCAAAAGATCAGTCTG
ATP-binding casette, sub-family C, member1	ABCC1	CTCTATCTCTCCCGACATGACC	AGCAGACGATCCACAGCAAAA
ATP-binding casette, sub-family C, member2	ABCC2	TCTCTCGATACTCTGTGGCAC	CTGGAATCCGTAGGAGATGAAGA
ATP-binding cassette, sub-family G, member 2	ABCG2	ACGAACGGATTAACAGGGTCA	CTCCAGACACACCACGGAT
Nuclear factor (erythroid-derived 2)-like 2	Nrf2	TCCAGTCAGAAACCAGTGGAT	GAATGTCTGCGCCAAAAGCTG
NAD(P)H dehydrogenase, quinone 1	NQO1	GGGATCCACGGGGACATGAATG	ATTTGAATTCGGGCGTCTGCTG
Glutathione S-transferase pi	GSTP1	TGCAAATACATCTCCCTCATCTACA	CGGGCAGTGCCTTCACAT
Glutathione S-transferase mu 4	GSTM4	AGAGGAGAAGATTCGTGTGGA	TGCTGCATCATTGTAGGAAGTT
Glutathione S-transferase alpha 2	GSTA2	TACTCCAATATACGGGGCAGAA	TCCTCAGGTTGACTAAAGGGC
UDP-glucuronosyltransferase 1-6	UGT1A6	TGATCCTGGCTGAGTATTTGGG	TGGGAATGTAGGACACAGGGT
Heme oxygenase (decycling) 1	HMOX-1	TCTCTTGGCTGGCTTCCTTA	ATTGCCTGGATGTGCTTTTC
Sequestosome 1	SQSTM1	GCACCCCAATGTGATCTGC	CGCTACACAAGTCGTAGTCTGG
Activating transcription factor 4	ATF4	ATGACCGAAATGAGCTTCCTG	GCTGGAGAACCCATGAGGT
Nuclear factor I/A	NFIA	GCAGGCCCGAAAACGAAAATA	TTTGCCAGAAGTCGAGATGCC
Protein kinase C, alpha	PRKCA	GTCCACAAGAGGTGCCATGAA	AAGGTGGGGCTTCCGTAAGT
Ectodermal neural cortex 1	ENC1 (NRPB)	GCTGCTGTCTGATGCACAC	AGAGTTGCACTACCATGTCCT
Rho family GTPase 3	RND3	GCTCCATGTCTTCGCCAAG	AAAACTGGCCGTGTAATTCTCA
UDP-glucose ceramide glucosyltransferase	UGCG	GAATGGCCGTCTTCGGGTT	AGGTGTAATCGGGTGTAGATGAT
ATPase, class II, type 9A	ATP9A	AAGTCAACTCCCAGGTCTACAG	CGCTGGTTCTTTTCAACGATGA
charged multivesicular body protein 1B	CHMP1B	GAATGAGTGCGCGAGTCGAT	GGTCTTCAATGTCGCATCCAT
epiregulin	EREG	GGACAGTGCATCTATCTGGTGG	TTGGTGGACGGTTAAAAAGAAGT
glutamate-cysteine ligase, modifier subunit	GCLM	CATTTACAGCCTTACTGGGAGG	ATGCAGTCAAATCTGGTGGCA
glutamate-cysteine ligase, modifier subunit	TXNRD	CATTTACAGCCTTACTGGGAGG	ATGCAGTCAAATCTGGTGGCA
SRY (sex determining region Y)-box 9	SOX9	AGCGAACGCACATCAAGAC	CTGTAGGCGATCTGTTGGGG
potassium channel tetramerization domain containing 5	KCTD5	AACGAGACAGCAAAACATCGC	TGACCAACTGCTCGAACTTCC
BCL2-associated athanogene 3	BAG3	TGGGAGATCAAGATCGACCC	GGGCCATTGGCAGAGGATG
